# Two Drosophila model neurons can regenerate axons from the stump or from a converted dendrite, with feedback between the two sites

**DOI:** 10.1186/s13064-017-0092-3

**Published:** 2017-08-17

**Authors:** Kavitha S. Rao, Melissa M. Rolls

**Affiliations:** Department of Biochemistry and Molecular Biology, The Pennsylvania State University, University Park, PA 16802 USA

**Keywords:** Axon regeneration, Microtubule polarity, Laser surgery

## Abstract

**Background:**

After axon severing, neurons recover function by reinitiating axon outgrowth. New outgrowth often originates from the remaining axon stump. However, in many mammalian neurons, new axons initiate from a dendritic site when the axon is injured close to the cell body.

**Methods:**

*Drosophila* sensory neurons are ideal for studying neuronal injury responses because they can be injured reproducibly in a variety of genetic backgrounds. In *Drosophila*, it has been shown that a complex sensory neuron, ddaC, can regenerate an axon from a stump, and a simple sensory neuron, ddaE, can regenerate an axon from a dendrite. To provide a more complete picture of axon regeneration in these cell types, we performed additional injury types.

**Results:**

We found that ddaE neurons can initiate regeneration from an axon stump when a stump remains. We also showed that ddaC neurons regenerate from the dendrite when the axon is severed close to the cell body. We next demonstrated if a stump remains, new axons can originate from this site and a dendrite at the same time. Because cutting the axon close to the cell body results in growth of the new axon from a dendrite, and cutting further out may not, we asked whether the initial response in the cell body was similar after both types of injury. A transcriptional reporter for axon injury signaling, puc-GFP, increased with similar timing and levels after proximal and distal axotomy. However, changes in dendritic microtubule polarity differed in response to the two types of injury, and were influenced by the presence of a scar at the distal axotomy site.

**Conclusions:**

We conclude that both ddaE and ddaC can regenerate axons either from the stump or a dendrite, and that there is some feedback between the two sites that modulates dendritic microtubule polarity.

**Electronic supplementary material:**

The online version of this article (doi:10.1186/s13064-017-0092-3) contains supplementary material, which is available to authorized users.

## Background

In response to axon severing, many neurons have the capacity to regenerate this part of the cell. Classic axon regeneration involves signaling from the site of injury back to the cell body, followed by initiation of outgrowth from the remaining axon stump [1–3]. In some cases, particularly in the peripheral nervous system, regrowing axons may ultimately reconnect with targets to recover function [3].

Initiation of regeneration from a remaining axon stump has been observed in many types of neurons in vivo, including interneurons in the mouse spinal cord [4], interneurons in snails [5], motor neurons in *C.elegans* [6] and *Drosophila* [7] and sensory neurons in *C. elegans* [8] and *Drosophila* [9]. It is likely that in all of these scenarios an initial MAP kinase signaling cascade that includes Dual Leucine Zipper Kinase (DLK) is required to initiate regeneration, as it has been shown to be central in all cases where it has been tested [6, 7, 10, 11].

While regeneration from a remaining stump is the most commonly studied type of axon regeneration, in many systems when the axon is severed very close to the cell body (proximal axotomy) a new axonal process arises from the dendrites rather than the cell body or short axon stump. This was first described in reticulospinal neurons of the sea lamprey, a jawless fish, where axon removal resulted in extensive sprouting from dendrites [12], and the ultrastructure of these sprouts resembled that of axons [13]. Similar observations have been made in mammalian neurons. Axon-like processes (ALPs) emerging from distal tips of dendrites after proximal axotomy have been reported in adult feline motoneurons [14–17] and interneurons [18]. Retinal ganglion cells in hamster [19] and spinal neurons in rats [20] also respond to proximal axotomy by sprouting axons from dendrites. Hippocampal neurons in dissociated and slice culture initiate growth of an axon from a dendrite after proximal axotomy, and in this case the new axon was shown to be able to form synapses [21]. More recently, regeneration of an axon from a dendrite was shown to occur after proximal axotomy in Drosophila sensory neurons [22]. This type of regeneration is therefore broadly conserved.

In addition to triggering regeneration from a dendrite, injury of the axon close to the cell body has the potential to induce formation of multiple axons. In feline spinal motoneurons, proximal axotomy often resulted in de novo axon growth from the cell body along with emergence of axons from dendrites [14, 15]. The most detailed description of this phenomenon is in cultured rodent hippocampal neurons, where injuries can be performed at very precise distances from the cell body. When the axon was severed within 35 μm of the cell body, neurons very rarely regrew exclusively from the stump, but fairly frequently initiated stump growth and growth from a dendrite. This “combined response” was only slightly less common than exclusive conversion of a dendrite to a growing axon [21]. When injuries were performed further from the cell body, only the stump regrew [21]. A similar spatial analysis of injury responses was performed in *C. elegans* DA/DB neurons, and here 30 μm from the cell body was also where the outcome of injury changed. When the stump was 30 μm or longer it was competent for regeneration, and when shorter a new axon emerged from the cell body [23].

**Fig. 1:**
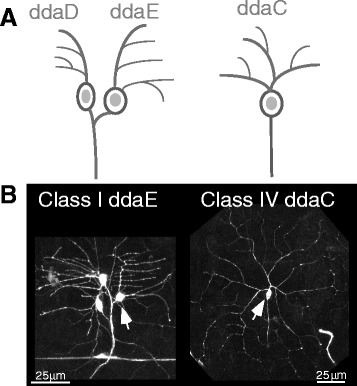
Neurons used in this study. **a** Schematics of the two larval Class I dendritic arborization neurons in the dorsal cluster are shown on the left, and a similar schematic is shown for the Class IV neuron in the cluster. **b** Confocal images of dorsal Class I and Class IV neurons expressing EB1-GFP are shown. The Gal4-driver used for Class I neurons is 221 and for Class IV is 477. The cell bodies of the ddaE and ddaC neuron are indicated with *arrows*. Ventral is down and posterior is to the right in all images and schematics

We have been using Drosophila sensory neurons as a genetically tractable model system in which to study axon regeneration. However, the basic responses of different sensory neurons to proximal and distal axotomy have not been comprehensively analyzed. Drosophila larval sensory neurons are particularly appealing as a system for studying neuronal injury responses because their cell bodies, dendrites and proximal axon are directly under the cuticle and epidermal cells on the body wall and so are optically accessible in whole animals. Drosophila dendritic arborization neurons are non-ciliated sensory neurons that can be classified into four groups based on complexity of their dendrite arbor [24]. Within these general classes, each neuron can be identified by position in each segment of the animal. Two identified neurons have been used in most of the studies on axon regeneration: ddaE is a Class I (simple) neuron and ddaC is a Class IV (complex) neuron (Fig. 1). The ddaE neuron can regenerate from a dendrite after proximal axotomy [22], but has been reported not to be able to perform classic axon regeneration from the stump after more distal axotomy [25]. In contrast the ddaC neuron regenerates from the axon stump after distal axotomy [9, 25, 26], but has not been tested for regeneration after proximal axotomy. It is also not known whether either of these model cells can generate multiple axons after proximal injury.

In this study we set out to do two things. First, we aimed to complete our basic understanding of the regenerative abilities of the model neurons ddaE and ddaC. Second, we asked whether the remaining axon stump influences the events that convert a dendrite to a new axon. In contrast to a previous study, we show that the Class I ddaE neuron can regenerate an axon from a stump, and therefore has the capacity to do both types of axon regeneration. In addition, we show that the Class IV ddaC neuron can regenerate an axon from a dendrite, and thus also has the complete complement of axon regenerative abilities. We also show that, in Drosophila, as in vertebrates, axons can grow from multiple sites after proximal axotomy. We also identify a novel feedback mechanism between the two regenerating axons, such that when a scar blocks re-growth from the axon stump, the microtubule polarity based changes in the dendrite that converts to an axon become accelerated.

## Results

### Axons regenerating from dendrites grow along the nerve when they encounter it

Drosophila sensory neurons can be injured precisely using a pulsed UV laser, and regeneration can be tracked in living larvae over several days [22]. Class IV ddaC neurons regenerate robustly from axon stumps after severing at a distance of 20 or more microns from the cell body [9, 25, 26]. In contrast, the Class I ddaE neurons have been reported not to regenerate from axon stumps after similar injury [25]. This result is somewhat confusing as ddaE neurons can convert a dendrite to a regenerating axon and grow on average several hundred microns by 96 h after injury [9, 22, 27], indicating that the cells can activate an axon regeneration program.

One possible explanation for the differential ability of the ddaE cell to regenerate from the stump and from the dendrite is that these types of regeneration are distinct in some way. We previously showed that axons regenerating from dendrites have axonal microtubule polarity and also exclude a dendritic marker [22], however, we were not able to demonstrate that they could follow axonal cues to lead them to their targets in the central nervous system (CNS) because their point of origination in the dendrite arbor is spatially removed from the nerve and the glial cells that wrap it. Therefore one potential difference between regeneration from the stump and the dendrite is glial vs. non-glial environment. Indeed in mammals, differences in glia are one factor believed to underlie poor regeneration in the CNS compared to the periphery [28].

**Fig. 2:**
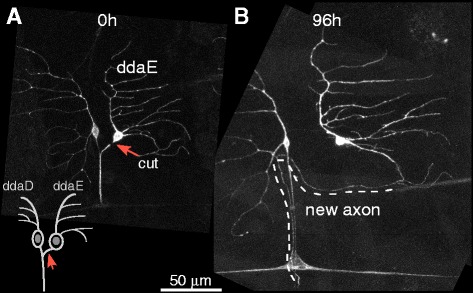
A new axon emerging from a dendrite can grow along the nerve. **a** The axon of a Class I ddaE neuron was axotomized very close to the cell body. The *orange arrow* shows the cut site, and a schematic is included. **b** The same neuron was imaged 96 h after injury. One of the dendrites converted to an axon and re-joined other axons in the same segment of the larvae. *Dotted line* shows the path of the new axon

To test the hypothesis whether contact with glia might influence ddaE regeneration, we collected many examples of axons regenerating from dendrites. After performing hundreds of proximal axotomies on ddaE neurons, we noted rare instances in which the axon growing out of a dendrite randomly encountered the glial wrapping of the nerve. When this happened, the regenerating axon grew along the nerve towards the CNS (Fig. 2). The position of the nerve is indicated by the axon of the other Class I neuron in this region visible with the 221-Gal4 driver. Although not visible, axons from other sensory neurons as well as motor neurons are part of this nerve. This ability to grow along the nerve is similar to that of ddaC neurons regrowing from the axon stump [9]. Thus the ability to use the nerve as a source of guidance cues is another similarity between axons regenerating from the stump and a dendrite. For axons regenerating from the stump the source of guidance cues could either be the other axons or the surrounding glia, while for axons regenerating from a dendrite it would most likely be the glia as the axons would contact the nerve from its outside.

### Class I ddaE neurons can regenerate from the axon stump

**Fig. 3:**
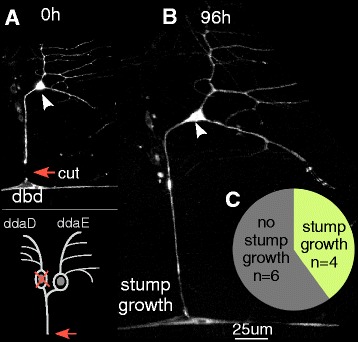
Class I ddaE neurons can regenerate from the axon stump. **a** An axon of the Class I ddaE neuron (cell body marked with an *arrowhead*) was severed 24 h after laser-mediated ablation of the neighboring Class I ddaD neuron. The site of injury (*orange arrow*) is located near the dorsal bipolar md neuron (dbd). **b** The same neuron was imaged 96 h after injury. Growth from the remaining axon stump was observed. **c** Out of 10 axotomized neurons, 4 neurons showed stump growth and 6 neurons did not grow from the stump

Because ddaE axons regenerating from a dendrite were not negatively affected by glial contact, we decided to retest the response of ddaE cells to distal axon severing. In the dorsal cluster of neurons that houses the ddaE cell, there is another Class I neuron, ddaD. All Gal4 drivers that express in ddaE also drive expression in ddaD. Axons from these two cells bundle with one another, and the nerve, 10–20 μm from the cell body. For initial tests on whether the ddaE neuron could regenerate from the stump, we wanted to eliminate the ddaD cell so that we could trace the ddaE axon in isolation. The ddaD neuron was therefore killed by aiming a pulsed UV laser at its nucleus, and the ddaE axon was severed 24 h later (Fig. 3a). In all cases the axon was severed at least 20 μm from the cell body, close to the position of the dorsal bipolar md neuron (dbd). Beyond this cell, the nerve dives below the surface and is more difficult to image at high resolution. We imaged the cell 96 h after severing to determine whether the axon stump had reinitiated growth (Fig. 3b). In 4 out of 10 cells tested, the stump was able to initiate growth indicating these cells are capable of regenerating after distal axotomy (Fig. 3c). Thus ddaE neurons can initiate axon regeneration from a remaining stump, although not in all individual cells. This inability of all cells to initiate regeneration from the stump likely explains the previous lack of regeneration in ddaE compared to ddaC neurons [25].

### Class IV ddaC neurons can regenerate from a dendrite after proximal axotomy

**Fig. 4:**
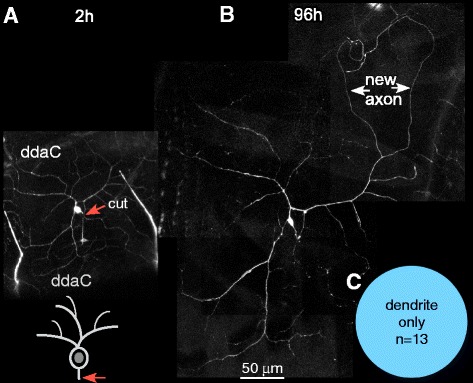
Proximal axotomy in Class IV ddaC neurons induces regeneration of an axon from a dendrite. **a** An axon of a Class IV ddaC neuron was axotomized very close to the cell body (*orange arrow*) and imaged 2 h after injury. **b** The same neuron was imaged 96 h after injury to track regeneration. *White arrows* indicate a dendrite that has been converted to an axon. The identity of the new axon was confirmed by its plus-end-out microtubule polarity. **c** All 13 axotomized neurons converted a dendrite to an axon following proximal axotomy

To complete our characterization of regeneration capabilities in Class I and Class IV neurons, we wanted to test whether Class IV ddaC neurons can regenerate axons from dendrites after proximal axotomy. Class IV ddaC neurons have been used extensively to study regeneration after distal axotomy [9, 25, 26]. However, it is not known whether the large and highly branched dendrite arbor can support conversion to an axon. We labeled Class IV ddaC neurons by expressing UAS-EB1-GFP under the control of the 477-Gal4 driver. Using a pulsed UV laser, the axon of a single ddaC neuron was severed close to the cell body (Fig. 4a). The same neuron was imaged 4 days post-injury to track regeneration (Fig. 4b). In all cases (*n* = 13), a dendrite converted to an axon and grew extensively (Fig. 4c). In most cases a single dendrite initiated growth, but occasionally more than one changed polarity and grew; no growth was observed from the remaining stump. We conclude that, like ddaE neurons, ddaC neurons can regenerate from either the axon stump or from a dendrite.

### New axons can grow from the stump and a dendrite in the same cell

So far we have shown that both Class I and Class IV neurons can regenerate from both the axon stump and dendrite after distal and proximal axotomy respectively. In our studies until this point, we had assayed regeneration either from the stump or the dendrite, but had not tested whether both might occur in the same cell. As injury near the cell body can lead to outgrowth from both sites in mammals, we wished to determine whether *Drosophila* neurons could also support multiple growth sites.

**Fig. 5:**
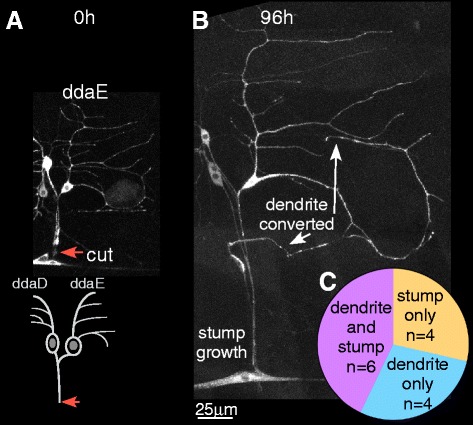
Distal axotomy often leads to formation of two axons in Class I ddaE neurons. **a** An axon bundle including the ddaE axon was axotomized at least 20 μm from the cell body (*orange arrow*). **b** The same neuron was imaged 96 h after injury. One of the dendrites of the Class I ddaE neuron converted to an axon as indicated by *arrows*, in addition to re-growth from the axon stump. **c** Out of 14 axotomized ddaE neurons, 6 grew from the stump and dendrite, 4 grew from the stump only and 4 grew from the dendrite only

As the experiments in which ddaD is killed to allow imaging of ddaE in isolation (Fig. 3) are somewhat cumbersome, we used an alternative approach to look for multiple sites of outgrowth. To clearly track regeneration from the stump, we severed axons of both ddaD and ddaE at the same point (Fig. 5a). That way, there was a clear gap in the axon bundle to allow tracking of subsequent stump growth. Out of 14 ddaE neurons examined, 4 neurons regenerated by converting a dendrite to an axon, 4 neurons regenerated from the original axon stump and 6 neurons regenerated from both the stump and dendrite (Fig. 5b-c). Axon growth was assessed by acquiring high magnification images of the region where the axons were cut. If the ddaD neuron initiated growth, but the ddaE did not, the blunt stump of ddaE was usually visible. In cases where clear images could not be acquired or there was ambiguity about outgrowth, the cells were not counted.

**Fig. 6:**
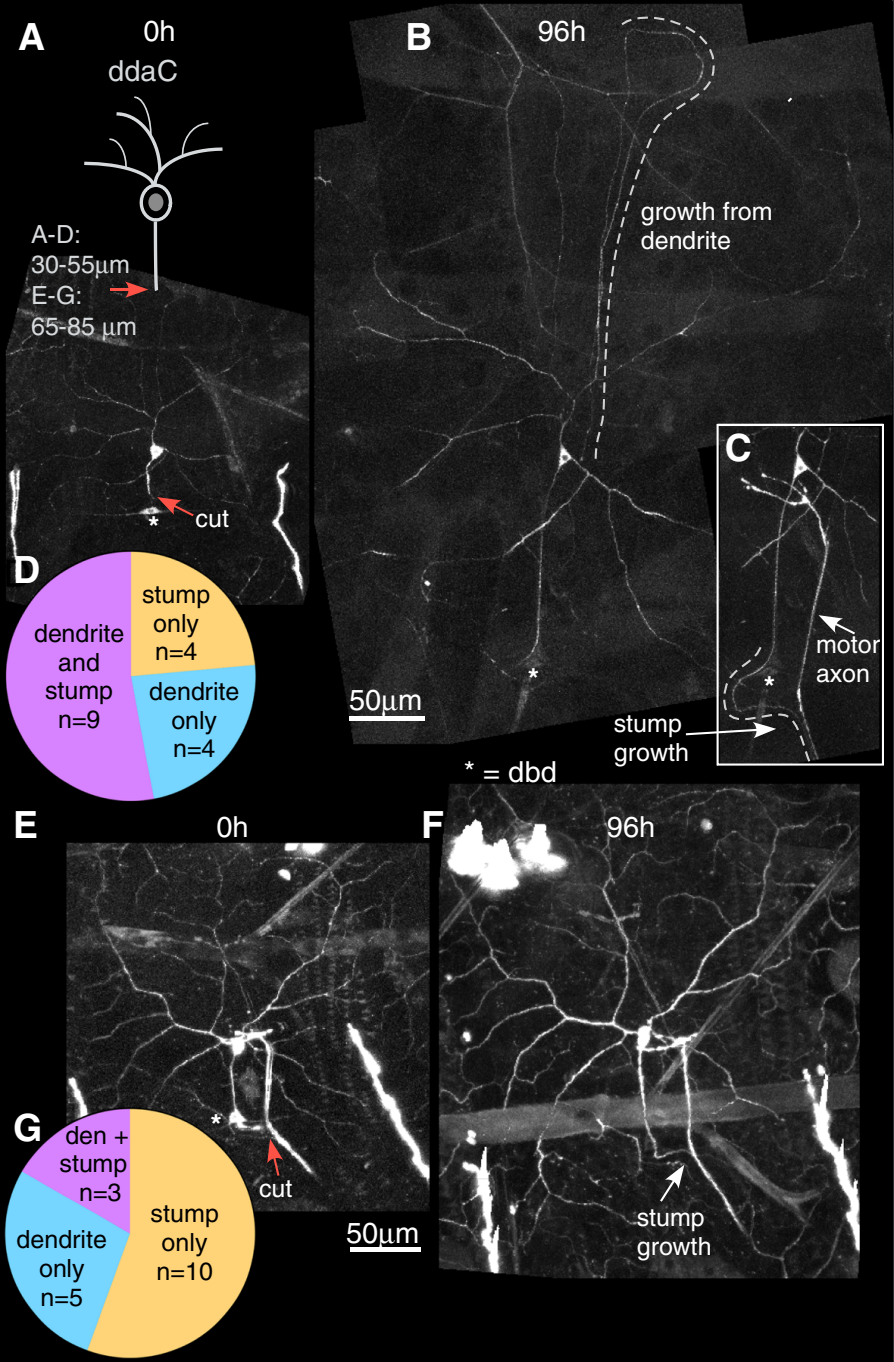
Distal axotomy often leads to formation of two axons from two locations in Class IV ddaC neurons. **a** An axon of a Class IV ddaC neuron was axotomized (*orange arrow*) more than 30 μm from the cell body. An asterisk indicates the dorsal bipolar md neuron (dbd). **b** The same neuron was imaged 96 h after injury. One of the dendrites converted to an axon as indicated by the *dashed line*. **c** The inset shows the same cell as in A and B, but at a different depth of focus to visualize the re-growing axon joining the nerve. **d** Out of 17 axotomized neurons, 4 neurons regenerated from the axon stump only, 4 neurons regenerated from the dendrite only and 9 neurons regenerated from both locations. **e** An axon of a Class IV ddaC neuron was severed at least 65 μm from the cell body; the cute site is indicated with an *orange arrow*. **f** The same neuron was imaged 96 h after injury. In this example, the remaining axon stump regenerated and joined the motor nerve bundle. **g** Out of 18 injured neurons, 10 neurons regenerated from the stump; 5 neurons regenerated from the dendrite and 3 neurons regenerated from both sites

Similar distal axotomy experiments were conducted in ddaC neurons labeled with EB1-GFP under the control of the 477-Gal4 driver (Fig. 6a). Of the 17 ddaC neurons that were distally axotomized, 9 neurons converted a dendrite to an axon in addition to regeneration from the stump, 4 neurons regenerated only from the stump, and 4 neurons regenerated by dendrite conversion only (Fig. 6b-d); dendrite conversion was assessed using microtubule polarity. In earlier studies looking at axon regeneration in ddaC neurons, a plasma membrane marker was used in most cases [9, 25, 26]. The membrane marker labels the entirety of the very complex arbor, including actin-based terminal branches. The marker used here, EB1-GFP, primarily labels regions of the dendrites where microtubules are concentrated. This makes identifying dendrite tips that have initiated growth more obvious. It is likely that in the previous studies dendrites were also initiating growth, but that it was missed.

In dissociated cultures of rodent hippocampal neurons, the ability to convert a dendrite into a regenerating axon was strongly reduced when axons were injured 35 μm or more from the cell body [21]. We therefore performed an additional set of distal axotomies on ddaC neurons, using the pulsed UV laser to cut 65 μm or more from the cell body (Fig. 6e). As in mammalian neurons, the number of neurons that converted a dendrite to an axon was reduced (although not eliminated) with additional distance from the cell body (Fig. 6f-g).

### The transcriptional response to injury is turned on with similar timing after proximal and distal axotomy

**Fig. 7:**
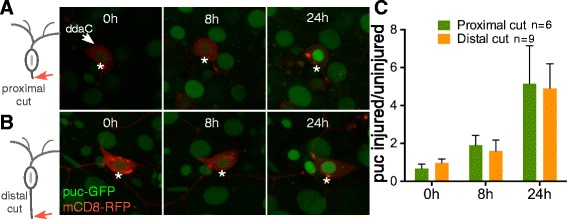
The post-injury JNK signaling pathway is turned on after proximal and distal axotomies. Class IV ddaC neurons expressing mCD8-RFP and puc-GFP were subjected to either proximal (*top*) or distal axotomy (*bottom*) as shown in the schematic diagrams. **a** Example images of nuclear puc-GFP signal after proximal axotomy are shown at the 0 h, 8 h and 24 h time-points. *Asterisks* indicate nuclei of the injured ddaC neurons. **b** Example images of puc-GFP signal after distal axotomy are shown at the 0 h, 8 h and 24 h time-points. **c** The graph shows the quantification of puc-GFP intensity in the nucleus of the injured ddaC neurons at different time-points, after normalization to the intensity of puc-GFP in the uninjured ddaC neuron in the neighboring segment of the same larva. Average intensities are shown in the bar graph, and error bars are standard deviations

Having shown that, like proximal axotomy, distal axotomy can lead to conversion of a dendrite to an axon in both cell types tested, we were interested in determining whether the events that precede regeneration were also similar in these two scenarios. In all model systems where it has been tested, a kinase cascade initiated by DLK is required to initiate the response to axon injury [6, 10, 11, 29]. In Drosophila, this kinase cascade works through the transcription factor fos to activate transcription [7], and one of the genes transcribed is the MAP kinase phosphatase puckered (puc) [7]. A puc-GFP reporter is a good readout of pathway activation [26]. To test whether the timing and approximate level of pathway activation was similar after proximal and distal axotomy, we performed these assays in animals that contained the puc-GFP reporter as well as a membrane marker. The ddaC axon was either severed adjacent to the cell body (proximal) or near the dbd neuron (distal), and images were acquired at 8 h and 24 h after injury (Fig. 7a-b). By 8 h after injury the puc-GFP signal in the nucleus of injured ddaC neurons was about twice as high as in uninjured ddaC neighbors, and by 24 h about five-fold higher (Fig. 7c). Similar increases were seen in cells with proximal and distal axotomy. Thus with this reporter, we did not detect a difference in timing or level of injury-induced transcription after the two types of injury.

### Conversion of a dendrite to axonal polarity is faster after proximal axotomy than after distal

Because injury-induced transcription and the ability to regenerate an axon from a dendrite were similar after proximal and distal axotomy, we considered the possibility that the only difference between these two types of injury is the presence of enough of an axon stump to support regeneration. If this were the case, stump regeneration would be entirely independent from regeneration from a dendrite. However, the probability that a dendrite will initiate growth is lower when the axon is cut further from the cell body, so there must be some feedback between the stump and the rest of the cell. The simplest model is that the two potential growth sites compete for resources at the time of outgrowth. To determine whether early feedback might exist, we analyzed microtubule changes required to convert a dendrite to an axon.

**Fig. 8:**
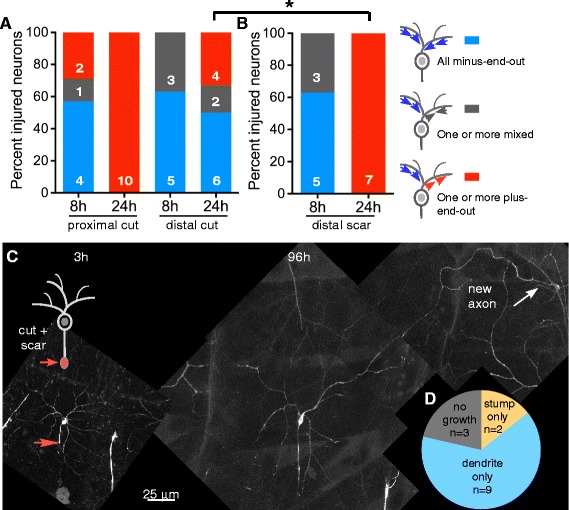
Conversion of a dendrite to axonal polarity is faster after proximal axotomy than after distal. **a** Class IV ddaC neurons were subjected to proximal or distal axotomy and microtubule polarity in the dendrites was tracked 8 h and 24 h after injury. Neurons with at least one plus-end-out process were placed in the *red* category. Neurons with one or more mixed processes were placed in the *grey* category. Neurons with all minus-end-out processes were placed in the *blue* category. The graph shows percentage of proximally or distally axotomized neurons in each category at 8 h and 24 h after injury. **b** Class IV ddaC neurons were subjected to distal scarring axotomy and microtubule polarity in the dendrites was tracked 8 h and 24 h after injury. Neurons were placed in the same categories shown in **a**. A Fishers Exact test between the red categories in A and B was performed for the 24 h time-points (*p < 0.05). **c** Example of a Class IV ddaC neuron subjected to distal scarring axotomy and imaged 3 h after injury. The *orange arrow* shows the cut site. The same neuron was imaged 96 h after injury. The axon stump failed to re-grow. A dendrite converted to an axon and showed extensive growth as indicated by *white arrow*. **d** Out of 14 axotomized neurons, 9 neurons converted a dendrite to an axon, 2 neurons grew from the remaining axon stump and 3 neurons failed to grow from either location

Dendrites convert to axons after proximal axotomy by disassembling their minus-end-out microtubule array and rebuilding it into a plus-end-out axonal array [22], so we assayed microtubule polarity after injury to probe early stages of the decision to convert a dendrite to an axon. Microtubule polarity was analyzed in Class IV ddaC neurons expressing EB1-GFP. EB1-GFP labels the plus-ends of growing microtubules, and so the movement of EB1-GFP comets denotes the orientation of microtubules [30]. Either proximal or distal axotomy was performed in the ddaC neuron, and EB1-GFP comets were analyzed in all dendrites at 8 h and 24 h post-axotomy (see methods). Injured ddaC neurons were then binned into one of three categories: 1) neurons with at least one plus-end-out neurite, 2) neurons with all minus-end-out neurites or 3) neurons with one or more mixed neurites (Fig. 8). At the 8 h time-point, in proximally axotomized neurons, 4 out of 7 neurons had all minus-end-out neurites, one cell had one or more mixed neurites and two had at least one plus-end-out neurite (Fig. 8a). At 24 h all proximally axotomized neurons had at least one plus-end-out neurite, suggesting that these neurons had completed specification of one of their neurites as an axon (Fig. 8a). In contrast, no distally axotomized ddaC neurons had a plus-end-out process 8 h after injury. Even 24 h post-axotomy, only one third of distally axotomized neurons had a plus-end-out neurite (Fig. 8a). Thus, although the puc-GFP injury reporter was activated with similar timing after proximal and distal axotomy, conversion of a dendrite to axonal polarity was more efficient after proximal axotomy. This difference in early conversion of a dendrite could perhaps account for the later difference in percent of neurons that initiate growth from a dendrite: 100% after proximal axotomy and about 50% after distal axotomy.

### Blocking regeneration from the axon stump accelerates microtubule polarity changes in the dendrite after distal axotomy

The difference in early steps in conversion of a dendrite to an axon after proximal and distal axotomy suggests that there is some signal that allows the cell to distinguish between the two injuries. One possibility is that this signal difference is due only to the position of the injury, and another possibility is that the capacity of the stump to regenerate suppresses regeneration from a dendrite. To distinguish between these two possibilities, we tested whether we could block regeneration of the stump after distal axotomy by generating more tissue damage at the injury site than in previous experiments. A scar was created at the injury site by prolonged use of the pulsed UV laser. Typically, the scar appeared as a bright, auto-fluorescent blob at the site of injury (Additional file 1: Figure S1B). On one occasion, the axon stump immediately retracted from the lesion site (Additional file 1: Figure S1A); in this case it is likely that the entire nerve, including all axons and surrounding glia, was severed. This particular cell did not regenerate, and is included in the results as one of the 3 in the set that did not exhibit regeneration (Fig. 8d). In all other cases the nerve remained connected across the scar.

After distal scarring axotomy 9/14 of injured neurons regenerated via dendrite conversion (Fig. 8c-d). Some neurons (3/14) failed to regenerate from either location and 2/14 of neurons regenerated from the axon stump (Fig 8d). Thus, distal scar axotomy reduced regeneration from the axon stump and frequently led to regeneration via conversion of a dendrite.

Next, we wished to examine whether blocking regeneration from the axon stump would affect microtubule polarity changes in the dendrite. To this end, we performed distal scarring axotomy in ddaC neurons and tracked movement of EB1-GFP comets at 8 h and 24 h post-axotomy. At the 8 h time-point, 5 out of 8 injured neurons had all minus-end-out neurites and the remaining 3 cells had one or more mixed neurites (Fig 8b), very similar to neurons with non-scarring distal injury. However, at the 24 h time-point, all injured neurons had at least one plus-end-out neurite (Fig. 8b), much like neurons after proximal axotomy. This change from initial similarity to distally injured axons to resemblance with proximally injured ones by 24 h implies that a feedback mechanism exists between the two regenerating axons, such that when regeneration from the stump is blocked, polarity reversal in a dendrite occurs more often.

## Discussion

Previous studies have shown that many vertebrate neurons have the capacity to regenerate from an axon stump when axons are injured at a distance from the cell body, or from a dendrite when the axon is injured closer to the cell body. Depending on the model, the distances that can be equated with close to the cell body and far vary, but this capacity seems quite general.

In this study, we demonstrate that the highly branched nociceptive ddaC neurons of *Drosophila* larvae respond to proximal axotomy by converting a dendrite to an axon (Fig. 3). This is similar to the response of much simpler ddaE proprioceptive neurons to proximal axotomy [22]. In both cell types the new axon that originates from a dendrite wanders around the body wall, and often grows away from the nerve that leads back to the CNS. There is thus some question about whether these processes are true axons [25]. We were able to reconcile this issue by showing that axons generated by dendrite conversion are able to recognize and grow along the nerve on the rare occasions they encounter it (Fig. 2). However, it is yet to be demonstrated if this new axon is able to re-innervate targets in the brain.

An interesting outcome of proximal axotomy in vertebrate systems is the formation of multiple axons [14, 15, 21]. In our study, we demonstrate that multiple axons can form after axon loss in the *Drosophila* peripheral nervous system. However, unlike previous studies in vertebrates, two axons were generated in *Drosophila* sensory neurons after distal axotomy but not proximal axotomy. One possible reason for this difference may be the distance of the injury site from the cell body. In mouse hippocampal neurons, a distance of 35 μm from the cell body was considered as proximal [21]. In our studies, proximal axotomy was performed by severing very close to the cell body. Therefore, in most cases, the remaining stump is extremely short, which may make it completely incompetent for growth.

In both ddaC and ddaE neurons, growth from a stump and dendrite at the same time was quite common. It would be very interesting to determine the fate of the two regenerating axons. Since the axon re-growing from the remaining stump seems to retrace its original route towards the ventral nerve cord, it is possible that the axon arising from a dendrite may be eventually pruned or retracted to maintain the ‘one-axon’ rule that normally governs these cells. With the current larval regeneration assays, the animal pupariates before these observations would be possible, but with long-lived larvae [31] this might be testable in the future. Similarly, while we have now shown that both ddaC and ddaE neurons can be used to study early stages of regeneration from the stump or from a dendrite, long-lived larvae may be necessary to use these model cells to study reinnervation of targets.

Many interesting questions remain about the early stages of axon regeneration that can be investigated in this system. For example, it is not known why severing the axon close to the cell body triggers regeneration from a dendrite, and how a dendrite is converted to a new axon. Moreover, it is not clear how the cell chooses a dendrite to convert to an axon. We did not notice any particular pattern of selection in ddaC neurons, but in ddaE neurons the large dorsal comb-like dendrite is rarely selected [22]. One hypothesis to account for this bias is that the dendrite that is less complex might be easier to convert to opposite polarity. From this study, it is also apparent that the severity of injury can trigger different outcomes: when a scar is generated during injury, conversion of a dendrite to a new axon is much more efficient than injury without a scar. This observation suggests that not only the injury itself is monitored, but perhaps also the type of injury or whether or not an axon growing from a stump encounters an impediment. The nature of the signals that would report to the cell body about type of injury or initial ability of the axon to grow are not known.

## Conclusions

Drosophila sensory neurons with simple or complex dendrite arbors have two ways they can regenerate axons: from the remaining stump or by converting a dendrite to a new axon. This suite of responses is similar to those of injured mammalian neurons, meaning that Drosophila is a good model system for understanding conserved repair pathways. The decision to convert a dendrite to an axon is influenced not only by the position of axon injury, but also whether the axon stump encounters a growth obstacle.

## Methods

### *Drosophila* stocks

Injury assays in Class IV ddaC neurons were performed by crossing fly lines expressing UAS-EB1-GFP under the control of 447-GAL4, with *yw* flies. Injury assays in Class I ddaE neurons were carried out by crossing fly lines containing 221-Gal4, UAS-EB1-GFP transgenes with *yw* flies. The puc-GFP reporter line was generated as part of a protein trap screen [32].

### Live injury assays in *Drosophila* larvae

Adult male and female flies were crossed in bottles capped with 35 mm petri dishes containing standard Drosophila media (food caps) and crosses were maintained at 25 degrees Celsius. Food caps with embryos on them were removed from the cross bottle every day and transferred to 10 cm petri dishes and incubated at 20 degree Celsius. Axotomy assays were performed on 3-day old larvae (3 days of incubation at 20 degrees Celsius) by placing them between a slide and coverslip and secured with tape. A MicroPoint pulsed UV laser system (Andor) was used to sever the axon. For proximal axotomy, the axon was severed very close to the cell body, while being careful to ensure no damage was done to the cell body. For distal axotomy, the axon was severed near the region where the bipolar neuron is situated (20–55 μm from the cell body).

Distal scar axotomy was performed similar to distal axotomy, but a scar was created by prolonged use of the pulsed UV laser. Far distal axotomy was performed by severing the axon 65–85 μm from the cell body. Images were taken immediately after injury (0 h) for proximal axotomy and 2-5 h after distal axotomy to check for complete severing. Regeneration was assessed 96 h after injury. The ddaD neuron was ablated by focusing the pulsed UV laser at the nucleus and the ddaE axon was severed 24 h after elimination. All images were acquired using a Zeiss LSM510 confocal microscope (Carl Zeiss). Z-projections were assembled using ImageJ software. Due to the elaborate branches of Class IV ddaC neurons, images of smaller regions were stitched together using Canvas software (ACD Systems).

### Microtubule polarity assay

Microtubule plus-ends were visualized with EB1-GFP in injured neurons at 0 h, 8 h, 24 h or 96 h time-points after axon injury. Time-lapse movies were acquired on the Zeiss LSM510 confocal microscope using a 40× objective at a frame of two images per second. Movement of comets was tracked in all dendrites. Comets were counted manually in ImageJ. Only comets that were in focus for at least three consecutive frames were considered. A neurite was defined as plus-end-out if at least 3 out of 4 comets moved away from the cell body. A neurite was defined as mixed if the number of comets moving towards and away from the cell body was between the thresholds for plus-end-out and minus-end-out. A neurite was defined as minus-end-out if at least 3 out of 4 comets moved toward the cell body. The percentage of neurons with at least one plus-end-out neurite, or mixed or minus-end-out neurites was then plotted using GraphPad Prism 6 software.

### puc-GFP reporter assay

Injury-induced activation of the JNK signaling pathway was visualized by crossing flies expressing 477-Gal4, UAS-mCD8-RFP with a puc-GFP protein trap line. Proximal axotomy or distal axotomy of Class IV ddaC neurons were performed and images were acquired immediately after injury (0 h) and 24 h after injury. Nuclear GFP intensity of the injured Class IV ddaC neuron was measured using ImageJ and normalized to nuclear GFP intensity of a neighboring uninjured Class IV ddaC neuron. Average nuclear GFP intensity in the injured/uninjured cells are plotted at each time point using GraphPad Prism 6.

## Additional file

Additional file 1: Figure S1. Example of distal scarring axotomy. (A) An example of a Class IV ddaC neuron subjected to distal scarring axotomy with a retracted axon stump, as indicated by the arrow, is shown. (B) An example of a Class IV ddaC neuron subjected to distal scarring axotomy is shown. The arrow indicates the presence of a visible scar. (PDF 1369 kb)

## Abbreviations

ALPs: Axon like processes
CNS: Central nervous system
DLK: Dual Leucine Zipper Kinase

## References

[1] Liu K, Tedeschi A, Park KK, He Z (2011). Neuronal intrinsic mechanisms of axon regeneration. Annu Rev Neurosci..

[2] Rishal I, Fainzilber M (2014). Axon-soma communication in neuronal injury. Nat Rev Neurosci.

[3] Navarro X, Vivo M, Valero-Cabre A (2007). Neural plasticity after peripheral nerve injury and regeneration. Prog Neurobiol.

[4] Kerschensteiner M, Schwab ME, Lichtman JW, Misgeld T (2005). In vivo imaging of axonal degeneration and regeneration in the injured spinal cord. Nat Med.

[5] Murphy AD, Barker DL, Loring JF, Kater SB (1985). Sprouting and functional regeneration of an identified serotonergic neuron following axotomy. J Neurobiol.

[6] Hammarlund M, Nix P, Hauth L, Jorgensen EM, Bastiani M (2009). Axon regeneration requires a conserved MAP kinase pathway. Science.

[7] Xiong X, Wang X, Ewanek R, Bhat P, Diantonio A, Collins CA (2010). Protein turnover of the Wallenda/DLK kinase regulates a retrograde response to axonal injury. J Cell Biol.

[8] Wu Z, Ghosh-Roy A, Yanik MF, Zhang JZ, Jin Y, Chisholm AD (2007). Caenorhabditis elegans neuronal regeneration is influenced by life stage, ephrin signaling, and synaptic branching. Proc Natl Acad Sci U S A.

[9] Stone MC, Rao K, Gheres KW, Kim S, Tao J, La Rochelle C, Folker CT, Sherwood NT, Rolls MM (2012). Normal spastin gene dosage is specifically required for axon regeneration. Cell Rep.

[10] Yan D, Wu Z, Chisholm AD, Jin Y (2009). The DLK-1 kinase promotes mRNA stability and local translation in C. elegans synapses and axon regeneration. Cell.

[11] Shin JE, Cho Y, Beirowski B, Milbrandt J, Cavalli V, Diantonio A (2012). Dual leucine zipper kinase is required for retrograde injury signaling and axonal regeneration. Neuron.

[12] Hall GF, Cohen MJ (1983). Extensive dendritic sprouting induced by close axotomy of central neurons in the lamprey. Science.

[13] Hall GF, Poulos A, Cohen MJ (1989). Sprouts emerging from the dendrites of axotomized lamprey central neurons have axonlike ultrastructure. J Neurosci.

[14] Linda H, Risling M, Cullheim S (1985). ‘Dendraxons’ in regenerating motoneurons in the cat: do dendrites generate new axons after central axotomy?. Brain Res.

[15] Linda H, Cullheim S, Risling M (1992). A light and electron microscopic study of intracellularly HRP-labeled lumbar motoneurons after intramedullary axotomy in the adult cat. J Comp Neurol.

[16] Rose PK, MacDermid V, Joshi M, Neuber-Hess M (2001). Emergence of axons from distal dendrites of adult mammalian neurons following a permanent axotomy. Eur J Neurosci.

[17] Rose PK, Odlozinski M (1998). Expansion of the dendritic tree of motoneurons innervating neck muscles of the adult cat after permanent axotomy. J Comp Neurol.

[18] Fenrich KK, Skelton N, MacDermid VE, Meehan CF, Armstrong S, Neuber-Hess MS, Rose PK (2007). Axonal regeneration and development of de novo axons from distal dendrites of adult feline commissural interneurons after a proximal axotomy. J Comp Neurol.

[19] Cho EY, So KF (1992). Characterization of the sprouting response of axon-like processes from retinal ganglion cells after axotomy in adult hamsters: a model using intravitreal implantation of a peripheral nerve. J Neurocytol.

[20] Hoang TX, Nieto JH, Havton LA (2005). Regenerating supernumerary axons are cholinergic and emerge from both autonomic and motor neurons in the rat spinal cord. Neuroscience.

[21] Gomis-Ruth S, Wierenga CJ, Bradke F (2008). Plasticity of polarization: changing dendrites into axons in neurons integrated in neuronal circuits. Curr Biol.

[22] Stone MC, Nguyen MM, Tao J, Allender DL, Rolls MM (2010). Global up-regulation of microtubule dynamics and polarity reversal during regeneration of an axon from a dendrite. Mol Biol Cell.

[23] Gabel CV, Antoine F, Chuang CF, Samuel AD, Chang C (2008). Distinct cellular and molecular mechanisms mediate initial axon development and adult-stage axon regeneration in C. elegans. Development.

[24] Grueber WB, Jan LY, Jan YN (2002). Tiling of the Drosophila epidermis by multidendritic sensory neurons. Development.

[25] Song Y, Ori-McKenney KM, Zheng Y, Han C, Jan LY, Jan YN (2012). Regeneration of Drosophila sensory neuron axons and dendrites is regulated by the Akt pathway involving Pten and microRNA bantam. Genes Dev.

[26] Stone MC, Albertson RM, Chen L, Rolls MM (2014). Dendrite injury triggers DLK-independent regeneration. Cell Rep.

[27] Chen L, Nye DM, Stone MC, Weiner AT, Gheres KW, Xiong X, Collins CA, Rolls MM (2016). Mitochondria and Caspases Tune Nmnat-mediated stabilization to promote axon regeneration. PLoS Genet.

[28] Huebner EA, Strittmatter SM (2009). Axon regeneration in the peripheral and central nervous systems. Results Probl Cell Differ.

[29] Xiong X, Hao Y, Sun K, Li J, Li X, Mishra B, Soppina P, Wu C, Hume RI, Collins CA (2012). The Highwire ubiquitin ligase promotes axonal degeneration by tuning levels of Nmnat protein. PLoS Biol.

[30] Stepanova T, Slemmer J, Hoogenraad CC, Lansbergen G, Dortland B, De Zeeuw CI, Grosveld F, van Cappellen G, Akhmanova A, Galjart N (2003). Visualization of microtubule growth in cultured neurons via the use of EB3-GFP (end-binding protein 3-green fluorescent protein). J Neurosci.

[31] Miller DL, Ballard SL, Ganetzky B (2012). Analysis of synaptic growth and function in Drosophila with an extended larval stage. J Neurosci.

[32] Morin X, Daneman R, Zavortink M, Chia W (2001). A protein trap strategy to detect GFP-tagged proteins expressed from their endogenous loci in Drosophila. Proc Natl Acad Sci U S A.

