# Supplementary figures and images for: Two Drosophila model neurons can regenerate axons from the stump or from a converted dendrite, with feedback between the two sites
Source: Neural Dev. 2017 Aug 17;12:15. doi: 10.1186/s13064-017-0092-3 (PMC5561650; doi:10.1186/s13064-017-0092-3)

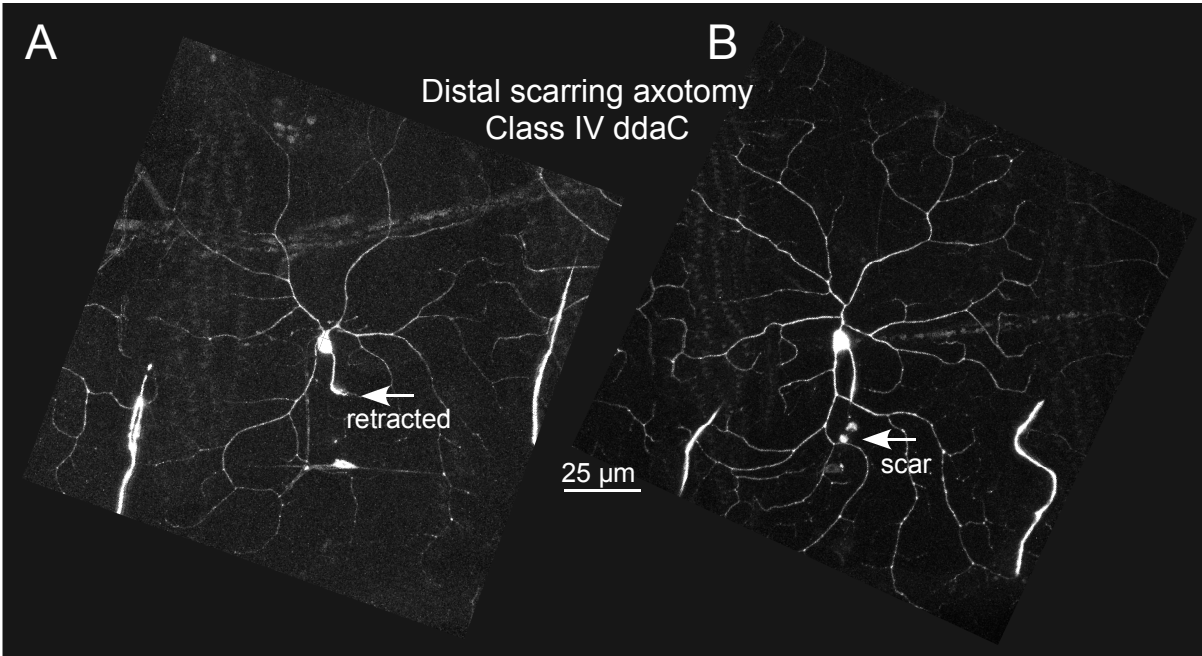

Supplement: Additional file 1: Figure S1. — Example of distal scarring axotomy. (A) An example of a Class IV ddaC neuron subjected to distal scarring axotomy with a retracted axon stump, as indicated by the arrow, is shown. (B) An example of a Class IV ddaC neuron subjected to distal scarring axotomy is shown. The arrow indicates the presence of a visible scar. (PDF 1369 kb) [file 13064_2017_92_MOESM1_ESM.pdf]
